# Surface Crack Detection in Precasted Slab Track in High-Speed Rail via Infrared Thermography

**DOI:** 10.3390/ma13214837

**Published:** 2020-10-29

**Authors:** Zai-Wei Li, Xiao-Zhou Liu, Hong-Yao Lu, Yue-Lei He, Yun‐Lai Zhou

**Affiliations:** 1 School of Urban Rail Transportation, Shanghai University of Engineering Science, Shanghai 201620, China; lzw_5220964@163.com (Z.-W.L.); lhy@sues.edu.cn (H.-Y.L.); hyldoc@163.com (Y.-L.H.); 2 College of Urban Transportation and Logistics, Shenzhen Technology University, Shenzhen 518118, China; liuxiaozhou@sztu.edu.cn; 3 State Key Laboratory for Strength and Vibration of Mechanical Structures, School of Aerospace Engineering, Xi’an Jiaotong University, Xi’an 710049, China

**Keywords:** infrared thermography (IRT), high-speed railway (HSR) track slab, surface crack, finite element (FE) simulation, field test

## Abstract

The surface crack of ballastless track slab can seriously reduce the serviceability and durability of high-speed railway (HSR). Aiming at accurately and efficiently detecting the slab cracks, this research proposes an infrared thermography (IRT)-based method for the surface crack, which is the most serious and common crack type in track slab. A three dimensional finite element (FE) model of IRT detection of concrete slab with surface cracks is established. The relation between the width of detectable cracks and the ambient temperature can be thereby obtained by inputting the measured thermodynamic parameters in the model. Parametric study shows that with ambient temperature higher than 15 °C, cracks with a width of no less than 0.2 mm can be well detected. Scale model test and field test are conducted, IRT method can effectively locate the slab surface cracks with width as small as 0.14 mm when ambient temperature is no less than 20 °C.

## 1. Introduction

**Figure 1:**
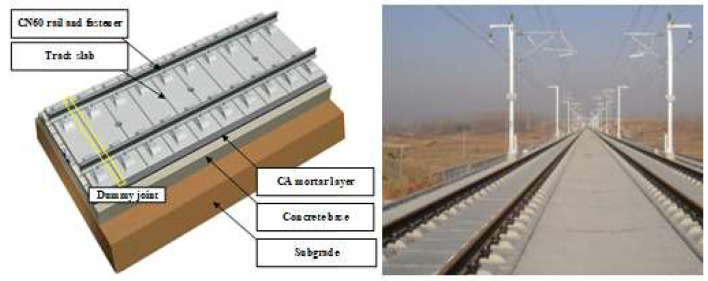
Longitudinally coupled prefabricated slab track (LCPST).

Longitudinally coupled prefabricated slab tracks (LCPSTs) are widely used in the new high-speed railway (HSR) lines in many countries, such as Germany, Japan, and China. In China, for instance, it accounts for about 35% of the total length of the national HSR network. LCPST is a multi-layer structure composed by concrete track slab, cement asphalt (CA) mortar layer and concrete base. The concrete slab is prefabricated and dummy joints are adopted to prevent concrete crack and its expansion, as shown in Figure 1 [1,2]. However, the practical experience of HSR operation in various countries shows that with the increase of the service time of the rail line, the performance of the track slab deteriorates significantly, and structural defects such as cracks and deformation of track slab can inevitably appear gradually [3]. Under the combined effect of temperature, rainwater, and dynamic load of reciprocating train, these cracks can aggravate the concrete looseness and water seepage near the cracks, lead to the corrosion of the internal reinforcing bars, and significantly reduce the service durability and stability of track slab structure [4,5]. With the continuous increase, expansion and penetration of the cracks, the integrity of track slab can be damaged, which directly affects the dynamic response characteristics of track structure, and even becomes the main hidden danger seriously threatening the operation safety of HSR [6]. How to detect the crack of track slab timely and effectively has become an important problem to be solved in the maintenance of HSR ballastless track.

Most of the railway departments around the world adopt manual inspection to detect the cracks of track slabs. However, manual inspection is characterised with low efficiency and great reliance on personal experience [7]. In addition, the maintenance time of HSR lasts only 1 to 2 h, which implicates massive employees for the track maintenance of hundreds and thousands kilometers. That can also be serious interference to route safety management [8]. To tackle this problem, extensive studies focused on the issues of track slab crack.

Considering that ballastless track slab is a typical prestressed concrete structure, many previous studies adopt the regular contactless nondestructive testing (NDT) methods for track defect detection, including impact echo testing [9,10,11], ultrasonic testing [12,13], ground penetrating radar [14,15,16], acoustic emission testing [17,18,19], etc. Combined with relevant mechanical theoretical calculations, track defect can be analysed qualitatively and quantitatively. However, these efforts mainly focus on track slab inter-layer defects, rather than slab surface cracks which is a critical problem for LCPSTs. Current research on slab crack is generally based on fixed-point local detection. The characteristics of the test data are relatively complex, which requires more experience. Apart from the above NDT methods, computer vision techniques with artificial intelligence methods are also adopted in track inspection [8,20,21]. Certainly there are also studies focused on the material scale degradation subjected to extreme condition [22,23,24]. However, for such a complex inspection environment as slab ballastless track structure, lighting factors and noise factors such as rails, fasteners and other components will make it very difficult to accurately identify the cracks in the track slab on site. There is no accurate model to describe the mode of crack damage. These factors become the great obstacles.

Infrared thermography (IRT), as a novel NDT method, has been more and more widely used in civil engineering. This method explores the surface infrared radiation and surface temperature field, to test surface or interior damage with thermal imaging camera [25]. Currently, IRT has been used in crack detection of building structures [26,27,28] and bridges [29,30,31], but it is relatively new in track crack inspection [32]. Thermography is suitable for crack detection with width as small as 0.5 mm with flash thermography [27].

This research, based on the field investigation into the characteristics of surface crack on track slab, proposes an IRT-based crack detection method for LCPST, with the combination of theoretical analysis and experiment. The remainder of this paper is organised as follows: the distribution of ballastless track crack is discussed in Section 2; the theory of thermography and the calculation of track slab thermodynamics is presented in Section 3; finite element (FE) analysis of concrete track slab is given in Section 4, followed by laboratory test and the field test results, as presented in Section 5; the last section gives the brief conclusions.

## 2. Field Investigation of Slab Crack

The actual defect characteristics are fundamental to the application of the IRT-based detection method for slab tracks. However, there is little study dedicated to carrying out field investigation into the cracks on the in-service track slabs. With the assistance of China Railway Shanghai Group, this research conducts field investigation on a 158 km-long HSR line in East China. This line was built in 2010 with CRTS-II slab track. The operation speed of the line is 300 km/h and the main types of foundation are subgrade and simply supported beam bridge.

**Figure 2:**
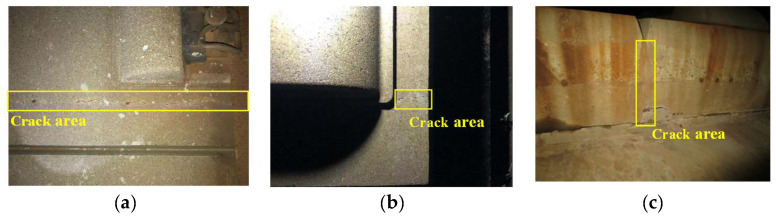
Track surface crack in the field: (**a**) Surface through crack; (**b**) Crack at sleeper; (**c**) Crack at dummy joints.

Figure 2 shows the typical slab cracks found by field investigation. The surface cracks can occur at dummy joints and other locations on the slabs. The cracks at dummy joints are mainly short cracks perpendicular to the side of the dummy joints and some through cracks expand through the surface and from side to bottom. The cracks at other locations on the slab are mainly short splayed diagonal cracks along the sleeper’s direction and transverse through cracks along the side of the sleepers. When the tensile stress of the track slab is greater than its tensile strength, the crack will occur at the weak location. Meanwhile, under the impact of periodic temperature load, the expansion and deformation of the track slab can happen continuously, which leads to the expansion of the crack along the transverse direction until it develops into a through crack.

Table 1 lists the statistical analysis results of the field investigation. A total of 73 slab cracks are identified. Among them, the proportions of cracks at dummy joints and other locations in bridge section are 52.5% and 47.5%, respectively, while the proportions in subgrade section are 51.6% and 48.4%, respectively. In both bridge section and subgrade section, the average values of width and length of the cracks at dummy joints are slightly larger than those of cracks at other locations, which is consistent with the design theory of ballastless track [33]. A total of 11 through cracks are found in subgrade and bridge sections, and the average width of through cracks is significantly larger than that of non-through cracks, with the maximum width of 0.82 mm. Compared with the non-through cracks whose average width is only about 0.13 mm, the damage state of through cracks is more serious. Referring to the management rule for China HSR [34], when surface crack width is larger than 0.2 mm, immediate repair is required.

## 3. IRT Testing for Slab Crack Detection

**Figure 3:**
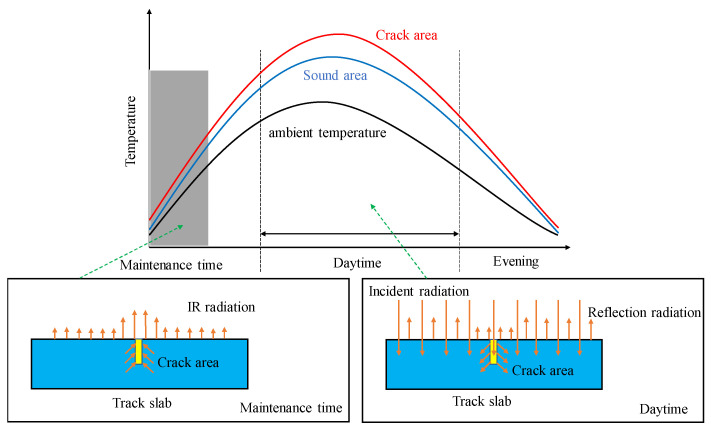
Mechanism of infrared imagery technology.

Any object with a temperature higher than absolute zero will continuously radiate infrared light to the external environment. Because the surface temperature and thermal physical parameters of the objects are different, the ability of the objects to radiate infrared light to the external environment is also different. The higher the temperature of the object, the stronger the ability of infrared radiation to the external environment. IRT, as a novel NDT technique, can detect the different intensity infrared thermal waves emitted by object surface and convert them into infrared thermal images with different colours [25]. For track slab, when a crack appears on its surface, a significant difference between the thermal conductivity of air medium in cracks and that of concrete materials will generate. When the heat flow enters the crack, the heat will accumulate in the crack area; when the heat flow exits, the air medium in cracks slows down the heat dissipation, resulting in the temperature difference of crack area and non-crack area, which generates temperature gradient field (see Figure 3). With IRT, we can detect the surface crack of track slab by collecting such temperature difference.

For the concrete track slab, in the actual heat transfer process, due to the uneven distribution of the internal temperature field, the following assumptions are made for the convenience of presenting the principle of heat sensor detection:

A. Due to the low thermal conductivity of concrete, it is considered that there is no coupling between temperature and structural deformation.
B. Track slab is assumed to be homogeneous and isotropic.
C. The thermal parameters of track slab materials are all constant and do not change with the change of temperature.

The heat transfer process inside an object can be expressed by the following formula [25] as

(1) $$\frac{\partial }{\partial x}\left(\lambda \frac{\partial T}{\partial x}\right)+\frac{\partial }{\partial y}\left(\lambda \frac{\partial T}{\partial y}\right)+\frac{\partial }{\partial z}\left(\lambda \frac{\partial T}{\partial z}\right)=\rho c\frac{\partial T}{\partial t}$$

where λ is the conductivity (W·m^−1^·°C^−1^), ρ is the density (kg·m^−3^), c is the specific heat capacity (J·kg^−1^·°C^−1^), t is time (s), T is temperature inside the slab (C), and x, y, and z are axis directions.

Since the window time HSR maintenance is always at night, the temperature field of track slab is mainly affected by the ambient temperature because of the continuous radiation and convection heat exchange between track slab and external environment. As a continuous concrete structure, the vertical dimension of track slab is far smaller than its transverse and longitudinal dimensions, so it can be treated as an infinite flat wall structure, and Equation (1) can be rewritten as

(2) $$\frac{\partial }{\partial x}\left(\lambda \frac{\partial T}{\partial x}\right)=\rho c\frac{\partial T}{\partial t}$$

To solve Equation (2), boundary conditions of temperature and heat transfer is established as

(3) $$s.t.\{\begin{array}{cc} T=T_{0} & ,t=0\\ \partial T/\partial x=0 & ,x=0\\ -\lambda \cdot \partial T/\partial x=h\left(T-T_{a}\right) & ,x=d \end{array}$$

where T is slab internal temperature (°C), λ is track slab conductivity (W/(m·℃)), ρ is slab density ($\mathrm{kg}/m^{3}$), c is specific heat (J/(kg·℃)), x is axis for thickness, d is track thickness (m), $T_{0}$ is the initial temperature (°C), $T_{a}$ is ambient temperature (°C), h is the integrated heat transfer coefficient (i.e., the sum of radiative coefficient $h_{r}$ and convective heat transfer coefficient $h_{w}$) ($W/\left(m^{2}\cdot ℃\right)$).

For dimensionless representation, let

(4) $$\{\begin{array}{c} \theta =T-T_{a}\\ F=\theta /\theta_{0}\\ X=x/d \end{array}$$

Substitute Equation (4) to Equations (2) and (3) yield

(5) $$\partial F/\partial \left(\mathrm{at}/d^{2}\right)=\partial^{2}F/\partial X^{2}\;s.t.\{\begin{array}{cc} F=F_{0}=1 & ,t=0\\ \partial F/\partial X=0 & ,X=0\\ \partial F/\partial X=\mathrm{hd}\cdot F/\lambda & ,X=1 \end{array}$$

where a is diffusivity coefficient (i.e., a=λ/ρc).

Use Fourier number $F_{o}$ to represent the time of unsteady heat conduction, and Bivot $B_{i}$ for the ratio of heat resistance between interior and surface of slab, dimensionless temperature can be given by

(6) $$\{\begin{array}{c} F_{o}=at/d^{2}\\ B_{i}=hd/\lambda \\ F=g\left(F_{o},B_{i},X\right) \end{array}$$

With slab surface crack, temperature difference between crack and non-crack area is given by

(7) $$\Delta T=T_{l}-T_{h}$$

where $T_{l}$ and $T_{h}$ refer to crack and non-crack temperature.

Combined with the influence of crack length (l) and width (w), the following equation is established

(8) $$\Delta T=g\left(\theta_{0},t,a,\;d,\;\lambda ,h,l,w\right)$$

According to π theorem and dimension harmony theorem, we select four basic parameters (i.e., ΔT, t, λ, and d), so that the dimensionless equation is given by

(9) $$F\left(\frac{\theta_{0}}{\Delta T},\frac{\mathrm{at}}{d^{2}},\frac{\mathrm{hd}}{\lambda },\frac{l}{d},\;\frac{w}{d}\right)=0$$

So ΔT can be expressed as

(10) $$\Delta T=f\left(\frac{\mathrm{at}}{d^{2}},\frac{\mathrm{hd}}{\lambda },\frac{l}{d},\;\frac{w}{d}\right)\times \theta_{0}=\Theta \times C\times R$$

where Θ is temperature coefficient mainly determined by ambient temperature, C is scale coefficient which is mainly determined by crack width, and R is the constant thermal coefficient.

Solving the temperature difference in Equation (10) is fundamental to the application of IRT in slab surface cracks detection. To determine the mapping relationship between the ambient temperature, crack width and temperature difference. Because of the irregular shape of the cracks on the surface of the slab and the randomness of the ambient temperature, it is impossible to express them with explicit analytical formula, which leads to the inaccuracy of the analytical solution of the temperature field.

## 4. Finite Element Analysis

### 4.1. Modeling

To effectively detect track slab cracks by IRT, parameters such as resolution, detection window length of the camera, ambient temperature, etc. need to be set. However, the field test of such parameters are very expensive and time-consuming [35]. Therefore, as an efficient and economic way, the FE method is widely used in the optimal design of IRT test parameters [35,36].

**Figure 4:**
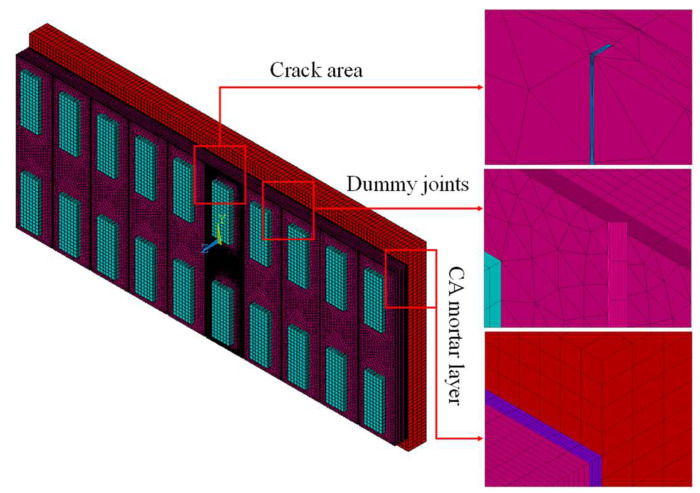
Three-dimension model for infrared thermography (IRT) of concrete track slab.

Based on the actual slab scale, a three-dimensional FE model of IRT detection of track slab with surface through cracks is established, in which Solid 70 element is used to simulate track slab, sleepers, CA mortar layer, and concrete base. The slab is 20 cm thick, 6.45 m long, and 2.55 m wide, supported by 10 sleepers (0.8 m × 0.3 m × 0.07 m), with V-shape dummy joints equally spaced between each sleeper at 0.65 m interval. The size of CA mortar layer and concrete base are 6.45 m × 2.55 m × 0.03 m and 6.45 m × 2.95 m × 0.3 m, respectively. Bonding treatment is adopted between layers to ensure connection. According to the investigation results in Table 1, through crack is set at the 5th sleeper. With Boolean operation, the actual irregular cracks are simplified as regular cracks with rectangular cross-section, and mesh refinement is adopted for crack area, as shown in Figure 4. The main calculating parameters are summarised in Table 2.

### 4.2. Setting of Thermal Parameters

From Equation (10), the initial slab temperature field and environmental meteorological parameters (solar radiation, ambient temperature and wind speed) need to be input in the simulation process. Based on the field investigation [37], thermal parameters (i.e., slab temperature) along the investigated line is collected at slab depth 0 mm, 100 mm, and 200 mm with sampling interval of 30 min for an entire year.

During the maintenance time of HSR line (i.e., 0:00 am–3:00 am), the ambient temperature range is between −3.6 and 28.3 °C, and it changes periodically with the seasons. With recognition of this, the ambient temperatures in simulation is set as −5, 0, 5, 10, 15, 20, 25, and 30 °C. Because the initial temperature field of the track slab is not evenly distributed and presents strong time-varying features, it is more suitable to use the measured average temperature of the track slab during the maintenance time as the initial temperature field. This is reasonable because there is no solar radiation effect, and the temperature difference between the track slab and the external environment is relatively small during this period.

Under the actual conditions, complex heat transfer process, including heat conduction, heat radiation, and heat convection can occur between the track slab and external environment. To simplify the model, all the heat effects generated by radiation heat transfer including solar radiation are considered to be converted into convective heat flow density, which is applied to the upper surface of the model as the boundary condition, and the side and bottom surfaces are assumed to be adiabatic. According to the management rules for Chinese HSR [34], crack width is set to be 0.1 mm, 0.2 mm, and 0.3 mm for parametric analysis.

### 4.3. Model Validation

**Figure 5:**
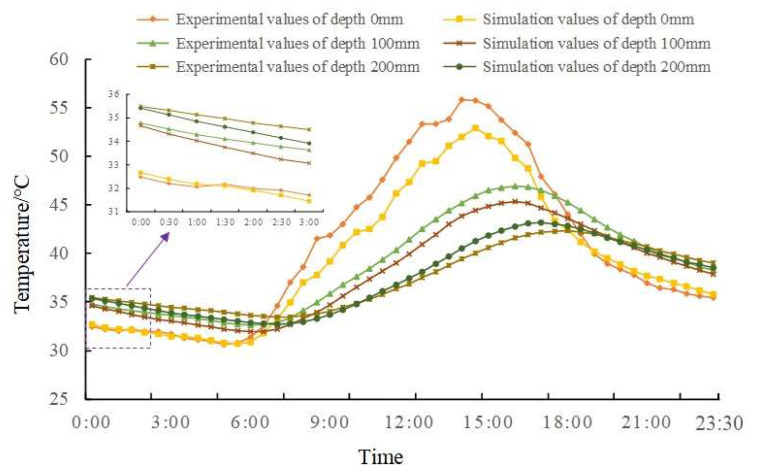
Comparison between the actual and simulated temperatures at varying slab depth.

Because the detection model established by the FE method is essentially a heat transfer model, this paper uses the measured data and the simulation results for comparative analysis to verify the model. The meteorological parameters and the initial temperature field parameters of the track slab, as listed in Table 3, are input into the model for calculation. Figure 5 shows the comparison of the actual measurement and simulation results of the internal temperature field of the track slab.

From Figure 5 that there is an error between the measured and the simulated value, especially at the slab surface (0 mm), and the maximum difference is 4.14 °C. This is due to the simplification of the boundary conditions in the FE model, in which the thermal effect caused by the direct solar radiation on the slab surface is ignored. During the period of window time for maintenance, the overall difference between the measured value and the calculated value in different depths is small, among which, the maximum difference between the two is only 0.25 °C on the slab surface and 0.58 °C at the depth of 200 mm, the boundary between track slab and mortar layer. The explanation for the difference at 200 mm is that the detection model simplifies the actual three-dimensional heat conduction process into one-dimensional and that the conductivity is different between slab and CA mortar layer.

### 4.4. Result Analysis

**Figure 6:**
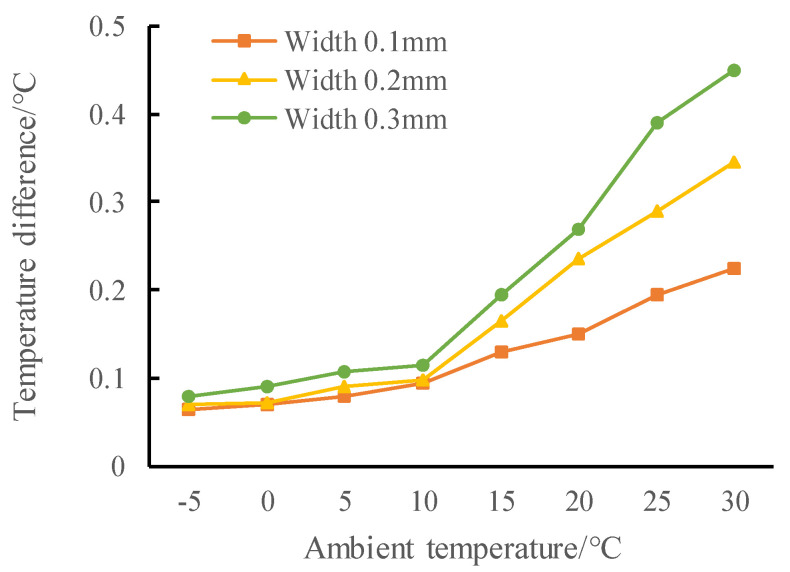
Crack-area temperature difference with varying crack width and ambient temperature.

The ambient temperatures (8 conditions) and crack widths (3 conditions), as mentioned in Section 4.2, are considered, and the results under 24 conditions are shown in Figure 6.

As ambient temperature and crack width increases, the temperature difference between crack and non-crack area shows a growing trend; the wider the crack width is, the greater the increase of the temperature difference will be. The larger the crack width is, the more air medium is filled in the crack. Under the influence of different thermal conductivity, the temperature effect of concrete and air medium is more obvious, and the temperature difference between crack area and non crack area is more prominent. When the ambient temperature is lower than 10 °C, due to the low thermal conductivity of the concrete, the overall temperature of the track slab is low, and the heat conduction effect between the crack area and the non crack area is slow. Even if the ambient temperature rises, the change of the temperature difference is not obvious, which is below 0.1 °C, and the temperature rise is relatively gentle, so in this range crack detection can be difficult and inefficient. When the ambient temperature is between 10 and 15 °C, through crack with width of no less than 0.2 mm can be detected. When the ambient temperature is higher than 15 °C, the temperature difference begins to rise obviously with the increase of temperature. At 30 °C, the temperature difference of 0.3 mm and 0.1 mm cracks reaches 0.45 °C and 0.23 °C, respectively, which means the performance of detection would be enhanced.

**Figure 7:**
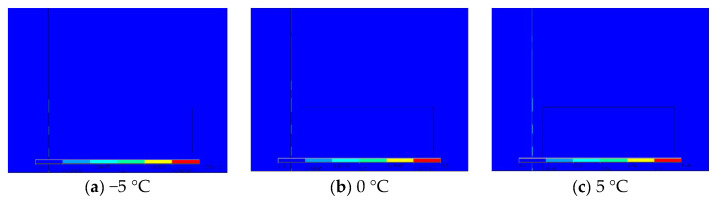

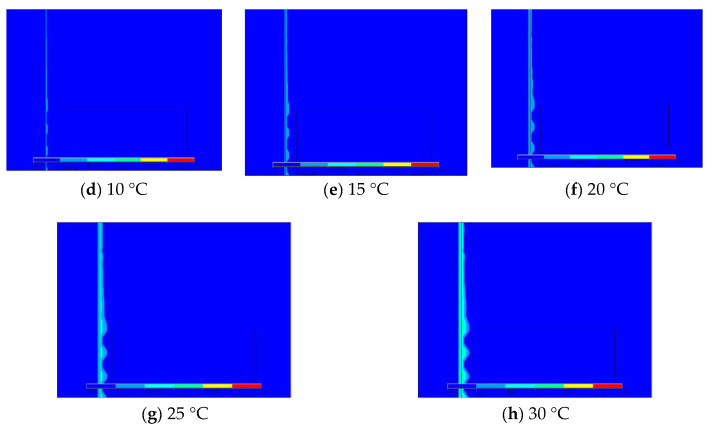
Temperature colour map for through crack with 0.2 mm width under different ambient temperatures.

Taking crack with 0.2 mm width as an example, Figure 7 shows the colour map of through crack under different temperatures. As ambient temperature increases, the temperature of the crack edge increases, and it spreads to the non crack area, resulting in a larger zone of detectable temperature area. Besides, under the influence of sleeper’s conductivity, the temperature difference zone along the crack direction becomes unstable on the side of the sleepers, while on the other side it is uniform.

To sum up, when the ambient temperature is more than 15 °C, the through cracks with a width of more than 0.2 mm can be detected.

## 5. Lab and Field Experiment

### 5.1. Laboratory Test

**Figure 8:**
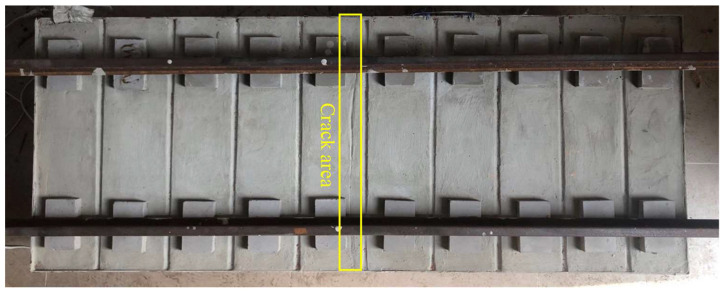
Scaled down sample of railway track.

To further determine the key parameters needed for IRT-based crack detection, samples with 1:5 scale are made in the laboratory for analysis. This is based on the similarity theory [38,39], which only needs to meet the conditions that the geometric size of track slab and crack is proportional, the number of Bivot is equal and the Fourier number is equal. The model size is 1290 mm (L) × 510 mm (W) × 200 mm (H), in which the size of the sleeper is 100 mm (L) × 60 mm (W) × 20 mm (H), and the dummy joint size is 1290 mm (L) × 14 mm (W) × 8 mm (d). The model material is determined according to the China railway design code [33]. The design strength grade of concrete is C55 and the cement is P·O42.5 ordinary Portland cement. The fine aggregate is natural river sand and the coarse aggregate composes of 80% natural gravel with diameter of 10–20 mm and 20% natural gravel with the diameter of 5–10 mm. The additive is hydroxy-acid water reducing agent, the admixture is fly ash, and the production water is ordinary tap water. An artificial through crack with a maximum width of 0.22 mm is prefabricated on the surface of the track slab. The test model is shown in Figure 8.

To ensure that the thermal imager has the same focal length and viewing angle during shooting, a metal bracket with height of 75 cm is fixed at the crack area, and the width of the upper structure of the metal bracket is suitable for the outer diameter of the lens of the thermal imager, with a length of 50 cm, to ensure that the thermal imager can cover the whole crack area during shooting. The thermal imager is inverted on the metal bracket, and the standard lens is used. The shooting range is about 6/10 of the surface area of the test model, the measurement temperature range is set to 15–35 °C, the focusing mode is set as auto focusing, and the thermal image display mode is set to rainbow mode for real-time storage.

**Figure 9:**
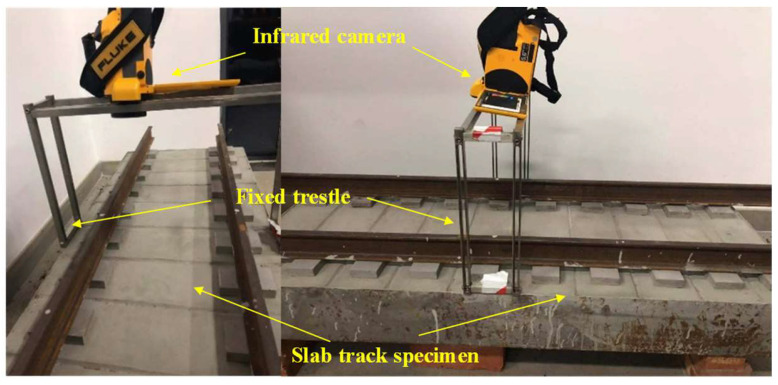
Infrared camera installed on the lab model.

The type of infrared camera is TIX620 by Fluke (Figure 9) with a heat sensitivity of 0.04 °C, spectral range of 7.5–14 µm, temperature measurement range of −40–600 °C, and display resolution of 1280 × 800. Generally, IRT can be passive and active. Passive detection relies on an external heat source while active detection relies on the ambient temperature [29]. To simulate the actual detection environment on HSR, this study uses the passive detection to carry out experiments. First, the test model is placed in an outdoor open space exposed to sunlight to simulate the heat transfer at daytime. The model is fully excited by active heat to ensure that the surface temperature field of the model produces a certain temperature gradient. During the test, the outdoor ambient temperature is 20.2 °C, the maximum solar radiation is 533 w/m^2^, and the average wind speed is 0.12 M/s. Two hours later, the model is moved indoor to simulate the passive surface crack detection under the condition of no heat radiation at night, as shown in Figure 9.

**Figure 10:**
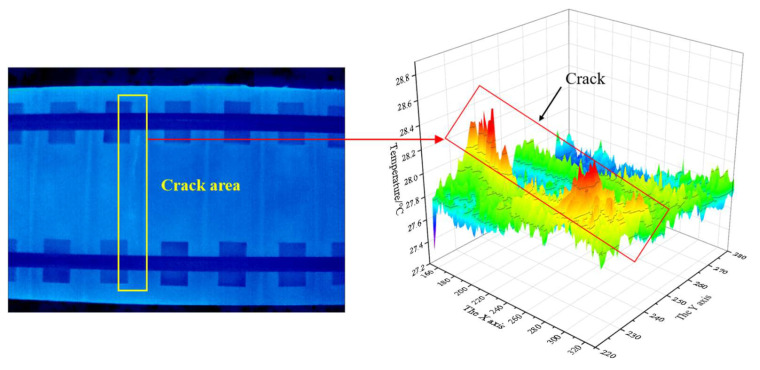
Thermography of lab experiment.

The test results are shown in Figure 10. The temperature field on the surface of the track slab presents an obvious non-uniform distribution among different structural components. The temperature field of surface crack area and non crack area is relatively uniform in their respective areas, but there are also some local hot spots and block areas. The heat transfer process of the track plate satisfies the three-dimensional heat transfer condition and the conduction rate is quite different under the actual conditions, and it is also affected by certain environmental noise and boundary convection. In the thermal image, heat concentrates along the crack direction, and expands to the surround areas, making the crack edge fuzzy. Correspondingly, on the thermography of the crack area (right panel figure), it also presents obvious fluctuation of the amplitude. The crack area shows discontinuous pattern with much more heat at the upper part while the lower part is similar to other areas. The actual size of the upper crack is larger than the design size, so that the temperature effect is strengthened, while the width of the lower crack is small, and the thermal effect is not obvious. Besides, in the heat transfer process, the lower crack is affected by local environment and concrete boundary, so that the heat balance speed is faster. The actual temperature difference in Figure 10 is far less than that in Figure 7, the result of FE simulation. To realise effective detection of track slab cracks under the complex effect of lab radiation and convection, the ambient temperature is not recommended to be less than 20 °C.

**Figure 11:**
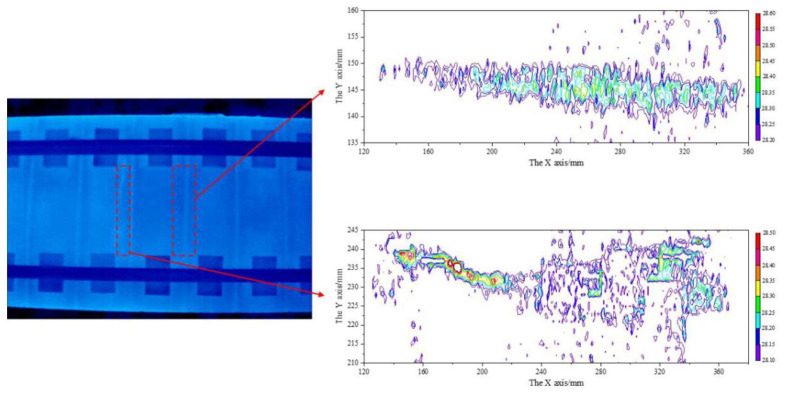
Temperature field isotherm at the slab surface.

To extract the feature of the surface crack area in Figure 10, we use the temperature field isotherm method [40] and the result is shown in Figure 11. At the surface crack area and the dummy joint area, there is a long high temperature envelope area in the isotherm diagram. The isotherm trend of the same dummy joint is roughly the same, only different in amplitude and background noise. By using the relative position relation and calculating the crack area according to the isotherm diagram, we can identify that the maximum crack width is 0.32 mm and the minimum crack width is 0.1 mm. Through the multi-point sum calculation, we can obtain the area value of 61.15 mm^2^, compared to the actual value of 66 mm^2^, with error of 7.35%. The performance is validated by applying IRT to crack detection with respect to crack area and width.

Under the complex conditions of lab experiment, the adoption of IRT for crack detection requires ambient temperature no less than 20 °C, instead the 10 °C from FE simulation. The applicability of IRT needs to be further discussed and analysed in the actual complex environment.

### 5.2. Field Test

The field test is carried out in an HSR (Sifang Ltd, Qingdao, China) section to detect the potential slab cracks using the same IRT equipment as in the laboratory test. During the test, the ambient temperature is 25.1 °C, the wind speed is 0.1 m/s, and the surface temperature of track slab is 32.78 °C.

**Figure 12:**
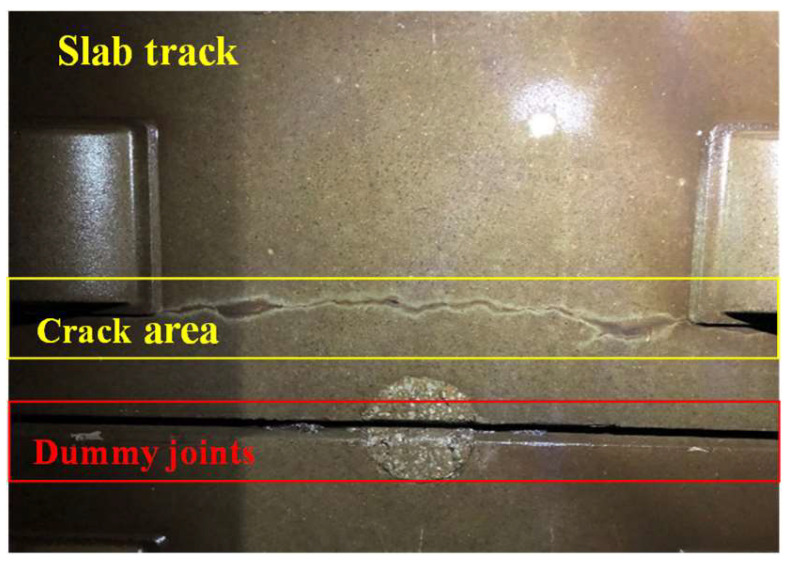
Field test for detection of track slab cracks.

**Figure 13:**
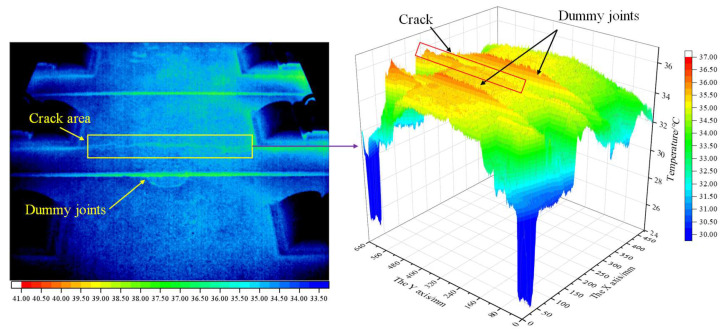
Typical thermography of track slab crack.

Figure 12 shows a typical through crack on the surface of track slab. The maximum, minimum and average crack width are 0.36 mm, 0.14 mm, and 0.27 mm, respectively. They are measured by a portable crack detector. The temperature range of the IRT equipment is set as 15–36.5 °C, and the shooting range is set as 3/10 of the track slab. Figure 13 shows the thermal image.

In Figure 13, different components of the track structure present different thermal images, and at dummy joints the image presents significant highlights, especially in the middle of the joint. With equal spacing, the effect of dummy joints can be easily removed before further identification of the surface cracks. In the actual crack area, the location is obvious, especially in the large-scale area, where the crack temperature and edge clearance are clear, and the characteristics are prominent.

**Figure 14:**
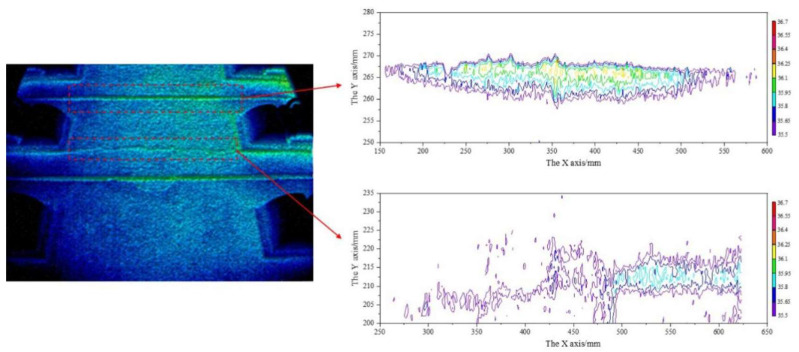
Temperature field isotherm of track slab crack.

**Figure 15:**
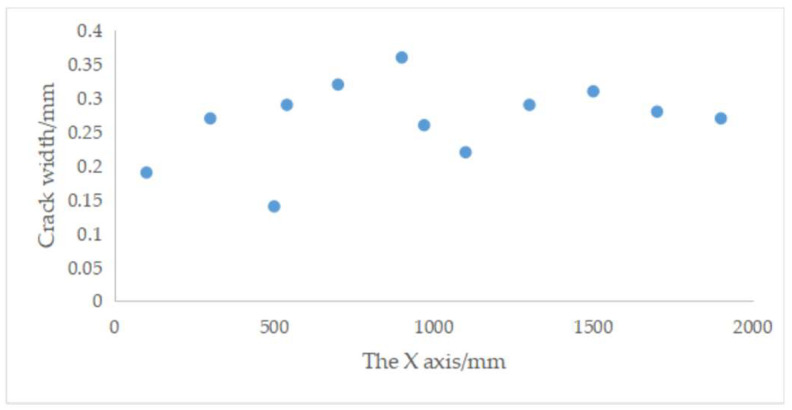
Results of crack width.

Figure 14 shows the temperature field isotherm. Figure 15 shows that the maximum, minimum and average values of crack width are 0.36 mm, 0.14 mm, and 0.267 mm, respectively, which is consistent with the actual crack width. Crack area is 521.88 mm^2^, 3.5% smaller than the actual value, which is 540 mm^2^. At high ambient temperature, it is feasible for the maintenance-of-way department to detect the slab cracks using IRT method.

## 6. Conclusions

This paper proposes an method for detecting the crack in the slab surface cracks in HSR with IRT detection. The mapping relation between the ambient temperature, crack width, and temperature difference is determined. A three-dimensional crack FE model for thermographing concrete slab track is established and is validated by the actual temperature field tests.

Through the scale-down model test in the laboratory and field test in HSR line, IRT method can effectively locate the slab surface cracks when ambient temperature is higher than 20 °C. FE parameter setting provides good reference to IRT actual application, but needs further validation in the field. Finally, a field test of IRT can detect the crack with width as small as 0.14 mm.

Although the width of the cracks on the surface of the slab track has been successfully detected, the depth of the cracks and the threshold of the detection temperature need further studies.

## Figures and Tables

**Table 1:** Statistics of the investigated track cracks.

Section	Location	Type	Count	Length/cm			Width/mm		
				Max	Min	Avg	Max	Min	Avg
Bridge	Dummy joint	Through cracks	2	/	/	/	0.36	0.14	0.27
		Others	19	16	12.11	15.26	0.19	0.1	0.16
	Others	Through cracks	3	/	/	/	0.32	0.11	0.24
		Others	16	18.8	2.89	14.94	0.13	0.02	0.10
Roadbed	Dummy joint	Through cracks	2	/	/	/	0.28	0.12	0.23
		Others	15	14.22	4	11.51	0.19	0.08	0.15
	Others	Through cracks	4	/	/	/	0.82	0.09	0.29
		Others	12	15.33	2.56	9	0.16	0.01	0.11

**Table 2:** The main parameters of track modeling.

Parameters	Slab	Sleeper	CA Mortar Layer	Concrete Base
Specific heat(J.kg^−1^·°C^−1^)	925	925	1350	925
Density(kg·$m^{-3}$)	2500	2500	1800	2300
Conductivity(W.m^−1^·°C^−1^)	3.23	3.23	0.261	3.23

**Table 3:** Meteorological parameters and the initial temperature field parameters.

**Meteorological Parameters**	Max solar irradiation (w/m^2^)	1136
	Max wind speed (m/s)	1.32
**Meteorological Parameters**	Depth/0 mm (°C)	32.49
	Depth/100 mm (°C)	34.77
	Depth/200 mm (°C)	35.49
